# Seasonal prevalence of malaria in West Sumba district, Indonesia

**DOI:** 10.1186/1475-2875-8-8

**Published:** 2009-01-09

**Authors:** Din Syafruddin, Krisin, Puji Asih, Sekartuti, Rita M Dewi, Farah Coutrier, Ismail E Rozy, Augustina I Susanti, Iqbal RF Elyazar, Awalludin Sutamihardja, Agus Rahmat, Michael Kinzer, William O Rogers

**Affiliations:** 1 Eijkman Institute for Molecular Biology, Diponegoro 69, Jakarta 10430, Indonesia; 2 Parasitic Diseases Program, Naval Medical Research Unit #2, Komp. P2P/PLP-LITBANGKES, Jl. Percetakan Negara No. 29, Jakarta Pusat 10560, Indonesia; 3 Department of Biomedicine and Pharmacology, National Institute for Health Research and Development, Jalan Percetakan Negara 29, Jakarta Pusat, 10560, Indonesia

## Abstract

**Background:**

Accurate information about the burden of malaria infection at the district or provincial level is required both to plan and assess local malaria control efforts. Although many studies of malaria epidemiology, immunology, and drug resistance have been conducted at many sites in Indonesia, there is little published literature describing malaria prevalence at the district, provincial, or national level.

**Methods:**

Two stage cluster sampling malaria prevalence surveys were conducted in the wet season and dry season across West Sumba, Nusa Tenggara Province, Indonesia.

**Results:**

Eight thousand eight hundred seventy samples were collected from 45 sub-villages in the surveys. The overall prevalence of malaria infection in the West Sumba District was 6.83% (95% CI, 4.40, 9.26) in the wet season and 4.95% (95% CI, 3.01, 6.90) in the dry. In the wet season *Plasmodium falciparum *accounted for 70% of infections; in the dry season *P. falciparum *and *Plasmodium vivax *were present in equal proportion. Malaria prevalence varied substantially across the district; prevalences in individual sub-villages ranged from 0–34%. The greatest malaria prevalence was in children and teenagers; the geometric mean parasitaemia in infected individuals decreased with age. Malaria infection was clearly associated with decreased haemoglobin concentration in children under 10 years of age, but it is not clear whether this association is causal.

**Conclusion:**

Malaria is hypoendemic to mesoendemic in West Sumba, Indonesia. The age distribution of parasitaemia suggests that transmission has been stable enough to induce some clinical immunity. These prevalence data will aid the design of future malaria control efforts and will serve as a baseline against which the results of current and future control efforts can be assessed.

## Background

Accurate information about the burden of malaria infection at the district or provincial level is required both to plan local malaria control efforts and to measure the impact of such efforts. Although many studies of malaria epidemiology, immunology, and drug resistance have been conducted at many sites in Indonesia [1-7], there is little published literature describing malaria prevalence at the district, provincial, or national level. Therefore, point prevalence surveys for malaria, designed to estimate malaria prevalence in the West Sumba District of East Nusa Tenggara Province, Indonesia, were conducted. Previous small scale surveys have identified individual villages in West Sumba with malaria prevalences ranging from 25 to 30% [8], although the sampling strategies are not fully described and it is unclear how representative these prevalences are. Therefore, two separate two stage cluster sampling surveys of malaria prevalence in the district were conducted, once in the wet season and once in the dry season, in order to obtain precise, district-wide estimates of malaria prevalence. These data will aid the design of future control efforts and will serve as a baseline against which the results of current and future malaria control efforts can be assessed.

## Methods

### Study site

Sumba is a member of the Lesser Sunda Archipelago, located in East Nusa Tenggara Province, Indonesia, at 9°40'S, 120°00'E. The island is divided into two districts, East Sumba and West Sumba. The population of West Sumba is approximately 400,000. Most residents are subsistence farmers. The climate is tropical, with a dry season from May to November and a wet season from December to April. Since completion of the study, West Sumba District has been divided into three separate districts.

### Study design and sampling strategy

The study consisted of two point prevalence surveys with two-stage cluster sampling. The primary unit of random selection was the sub-village, the smallest administrative unit for which there were census data. Selection of clusters used probability proportional to size (PPS) sampling, based on Government of Indonesia census data collected in 2005. Within each sub-village all residents of a set of houses chosen as follows were selected. Sumban villages are built around a central cluster of megalithic tombs. A pointer was spun in the village centre and the nearest house in the direction indicated by the pointer was visited. Beginning with this house, houses were visited in an expanding counterclockwise spiral until approximately 100 subjects were enrolled. At each household the participation of every individual who had slept in that house on the previous night was requested, and the sex and age of all individuals were recorded. Informed consent was obtained from subjects or their parents or guardians. The same set of 45 clusters was sampled twice, once in March 2007 (wet season) and once in August 2007 (dry season). Although the same 45 clusters were sampled in both surveys, no attempt was made to resample the same individual households in the clusters; instead a new random selection of households in each cluster was performed for the second survey.

### Human subjects research

The use of human subjects in these studies was approved by scientific and ethical review boards of the Naval Medical Research Unit #2, the Eijkman Institute, and by the Indonesian National Institute of Health Research and Development, and was conducted in accordance with regulations governing the protection of human subjects in medical research. Informed consent was obtained from all adult subjects and from the parents or legal guardians of minors.

### Data and sample collection

For each enrollee, the weight, height, and axillary temperature were measured, and inquiry was made if they had experienced fever in the previous 24 hours. All children between 2 and 9 years of age were examined for splenomegaly. Patients with signs and symptoms of malaria, including fever, chills, malaise, fatigue or other systemic complaints were tested with the Parascreen Rapid Test for Malaria (Zephyr Biomedicals, Goa, India). If positive, the patient received a three-day course of artemesinin/lumefantrine according to Indonesian Ministry of Health (MoH) guidelines. From finger or heel prick blood samples haemoglobin concentration was measured with a Hemocue device, and thick and thin blood films for malaria diagnosis were prepared. All malaria smears were read the same day, and if positive, a medical team returned to the sub-village the next day with anti-malarial treatment according to the above MoH guidelines.

### Laboratory methods

Thick and thin blood films were stained with Giemsa and examined by a certified microscopist using 1000× oil immersion light microscopy. At least 200 ocular fields were read before a slide was considered negative. Parasite densities were counted as parasites per 200 leukocytes and reported as parasites/mm^3 ^assuming a white blood cell count of 8000/mm^3^. A second certified microscopist reviewed all positive smears and 10% of negative smears. A third certified microscopist reviewed discrepant results; the majority reading was considered definitive. There were 195 discrepant slides, or less than 10% of the total.

### Data analysis

All data were recorded on standardized case report forms, double entered into MS Access (Microsoft Inc., Redmond WA) and exported for analysis in STATA (StataCorp LP, College Station, TX, USA), SPSS (SPSS Inc, Chicago, IL, USA), and Mathematica 5.2 (Wolfram Research Inc, Champaign, IL, USA). All statistical tests were two-tailed and significance was defined as p < 0.05.

## Results and Discussion

Two two-stage, cluster sampling malaria prevalence surveys were conducted in March (wet season) and August (dry season) of 2007. In the first survey 45 sub-villages were sampled out of a total of 300 in West Sumba. Within each sub-village a mean of 19.2 households (S.D., 3.8; range, 11–27) were sampled and within each household, samples were obtained from a mean of 5.2 (S.D., 2.9) individuals per household, for a total of 4,480 subjects. A total of 567 subjects who resided in the sampled households could not be contacted or enrolled. In the second survey, in the same 45 sub-villages, a mean of 18.3 households (S.D., 3.5; range, 11–26) were sampled per sub-village with a mean of 5.3 (S.D., 2.9) individuals per household, for a total of 4,375 subjects. In this survey, 906 individuals resident in the selected households could not be enrolled. Overall, it was possible to enroll and sample 8,870 out of 10,343 residents of the selected households (86%). The age and sex breakdown of the enrolled subjects and the missing members of the selected households are shown in Table 1. The missing individuals were disproportionately working age males.

**Table 1:** Study subject demographics.

Age Range (years)	March 2007 Wet Season				August 2007 Dry Season			
	
	Enrolled		Missing		Enrolled		Missing	
	
	Count (%)	% Male	Count (%)	% Male	Count (%)	% Male	Count (%)	% Male
0 to <5	825 (18.4)	53.7	10 (1.8)	50.0	830 (19.0)	49.3	60 (6.6)	60.0

5 to <10	759 (16.9)	51.1	16 (2.8)	37.5	771 (17.6)	49.2	61 (6.7)	50.8

10 to <20	983 (21.9)	46.8	34 (6.0)	67.7	962 (22.0)	45.2	155 (17.1)	64.5

20 to <40	1034 (23.0)	41.9	99 (17.5)	69.7	957 (21.9)	39.0	222 (24.5)	66.7

40+	879 (19.6)	50.3	75 (13.2)	70.7	855 (19.5)	46.4	190 (21.0)	66.8

N.R.*	15 (0.3)	66.7	333 (58.7)	59.8	0 (0.0)		218 (24.1)	58.3

Total	4495 (100.0)	48.4	567 (100.0)	62.6	4375 (100.0)	45.6	906 (100.0)	62.8

* Not recorded.

Table 2 shows the overall prevalence of infection with *Plasmodium spp *in the two survey periods. The raw prevalences of malaria infection in the sampled subjects in the wet and dry season were 6.92% (95% CI 6.18, 7.66) and 5.03% (95% CI, 4.38, 5.68), respectively. The 95% confidence intervals for the two-stage cluster design were adjusted using a finite population correction for the first stage and Taylor series linearization to calculate the standard error. Confidence intervals corrected for the study design were approximately three-fold wider than the uncorrected intervals (Table 2). Two possible sources of bias in the prevalence estimates were considered. First, it is unlikely that the procedure for selecting households within a cluster, proceeding outward in a spiral from a randomly chosen house near the centre of the sub-village, was completely random. It is possible that houses distant from the sub-village centre were under-represented, and, if such more distant households had a different risk of malaria, these prevalence estimates could be biased. For two reasons the magnitude of such biases is likely to have been small. First, most sub-villages are quite compact, with all houses clustered around the central tombs. Second, for each household the distance from the house to the sub-village centre was measured, and no association was found between distance from the centre and malaria prevalence.

**Table 2:** Malaria prevalence

Species	Wet Season Prevalence % (95%CI)	Dry Season Prevalence % (95% CI)
Any species (raw)	6.92 (6.18, 7.66)	5.03 (4.38, 5.68)

Any species (corrected for design)	6.92 (4.50, 9.34)	5.03 (3.05, 7.01)

Any species (weighted for missing subjects)	6.83 (4.40, 9.26)	4.95 (3.01, 6.90)

*P. falciparum* (weighted)	4.88 (2.95, 6.82)	2.88 (1.67, 4.08)

*P. vivax* (weighted)	2.16 (1.41, 2.90)	2.14 (1.19, 3.09)

*P. malariae* (weighted)	0.12 (0.03, 0.22)	0.09 (0, 0.23)

Another potential source of bias is introduced by the failure to recruit all members of a household. Approximately 14% of the members of selected households could not be contacted; if their risk of malaria differs from that of the enrolled subjects, the point estimates of malaria prevalence may be biased. An attempt was made to correct for such bias as follows. The subjects were divided into strata based on the sub-village, age group, sex, and season and each subject in each stratum was weighted by a factor equal to the inverse of the proportion of household members in that stratum which were not enrolled. Using this weighting is equivalent to assuming that missing subjects of a given age group and sex from a given sub-village have the same risk of malaria as the enrolled subjects of that age group, sex, and sub-village in the same season. Since the missing subjects were disproportionately adult males, and the prevalence of malaria was higher in children, this weighting slightly reduced the point estimate for malaria prevalence (Table 2). Similarly calculated estimates of prevalence for infection with *P. falciparum*, *P. vivax*, and *Plasmodium malariae *are shown in Table 2.

The prevalence of *P. falciparum *infection was higher in the wet season than in the dry (4.88% vs 2.88%), but there was little difference in the prevalence of *P. vivax *and *P. malariae *between seasons. Presumably, the higher prevalence of *P. falciparum *in the wet season is due to increased abundance of the vector mosquito species in this season. If a substantial fraction of *P. vivax *and *P. malariae *infections are due to relapse from the hypnozoite stage and chronic asymptomatic infection, respectively, then the lack of seasonality in these infections may be unsurprising.

**Figure 1:**
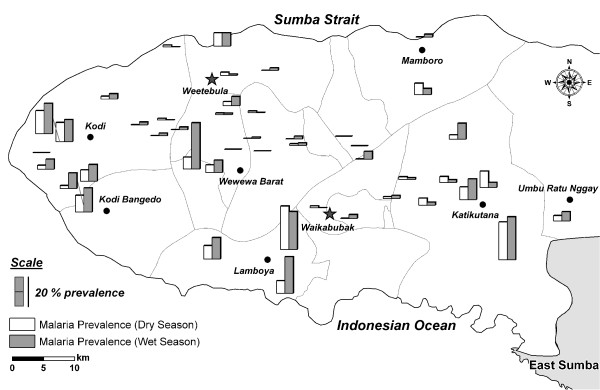
**Distribution of Malaria in West Sumba**. Shown is the distribution of *Plasmodium *infection prevalence (any species) in the 45 sub-villages sampled in the study.

**Figure 2:**
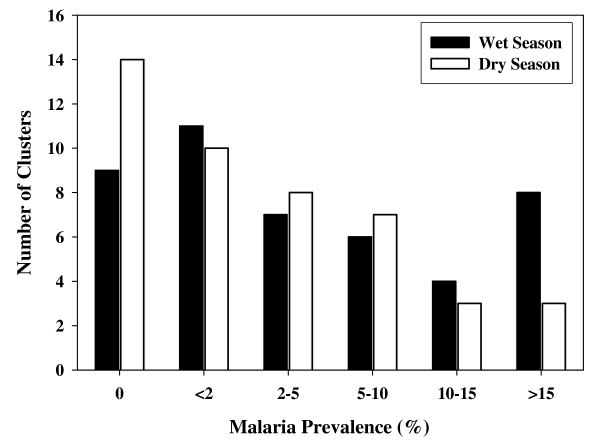
**Malaria Prevalence in Individual Sub-Villages**. Shown is a histogram of the number of sub-villages with malaria prevalence in the indicated ranges, in the wet and dry season surveys.

Malaria prevalence varied greatly between clusters and across West Sumba. Figure 1 shows the geographic distribution of malaria prevalence at the 45 sampled sub-villages in the wet and dry season. The sub-villages with the highest prevalence are mostly in the south-western part of the district. Figure 2 shows the distribution of malaria prevalences in individual clusters in the two seasons. The malaria prevalence in individual sub-villages ranged from 0–31% in the dry season and from 0–34% in the wet. In the wet season the number of sub-villages with no infections was lower, and the number of sub-villages with malaria prevalence >15% was greater, than in the dry season. The average difference in malaria prevalence between the wet and dry seasons in individual sub-villages was 2.4% (P = 0.0053, paired t test).

**Figure 3:**
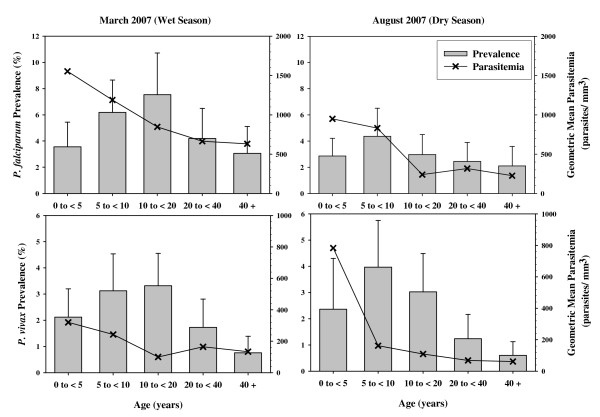
**Age Distribution of Prevalence and Parasite Density**. Shown are the age distributions of the prevalence of infection with *P. falciparum *and *P. vivax *and of the corresponding geometric mean parasitaemias among infected subjects in the wet and dry season surveys.

The age distribution of parasite prevalence and parasitaemia density provides suggestive information about the level of naturally acquired immunity to malaria and, indirectly, about the long term intensity and stability of malaria transmission. In populations not previously exposed to malaria who move to a malaria endemic area, for example recent Javanese migrants to Indonesian Papua, malaria is equally prevalent in all age groups [9]. On the other hand in populations stably exposed to malaria, prevalence and density of parasitaemia are higher in younger age groups [9,10]. In general, the higher the transmission intensity, the more rapidly immunity is acquired, and the younger the peak age for parasitaemia prevalence and density [11]. In this survey, malaria caused by *P. falciparum *and *P. vivax *was more common in children and teenagers than in adults in either the wet season or the dry season, and children with parasitaemia tended to have higher parasitaemia than adults. Figure 3 shows the age distribution of the prevalence and density of *P. falciparum *and *P. vivax *parasitaemia. The greatest prevalence of infection, for both *P. falciparum *and *P. vivax*, in either wet or dry season, occurred in subjects aged 5–20 years. The parasitaemia was highest in the youngest children and declined in the older age groups. The observed age distribution of prevalence and parasite density here is similar to that seen in meso-endemic areas of sub-Saharan Africa and in long-term migrants to Papua, and suggests that malaria transmission is sufficiently intense and stable to have induced at least partial clinical immunity. In Africa, young infants are protected by maternal antibodies [12]. Among 196 subjects six months or younger in age, only one was parasitemic. This very low prevalence in young infants is consistent with maternal immunity, but the possibility that social practices reduce the exposure of very young infants to mosquitoes cannot be excluded.

**Figure 4:**
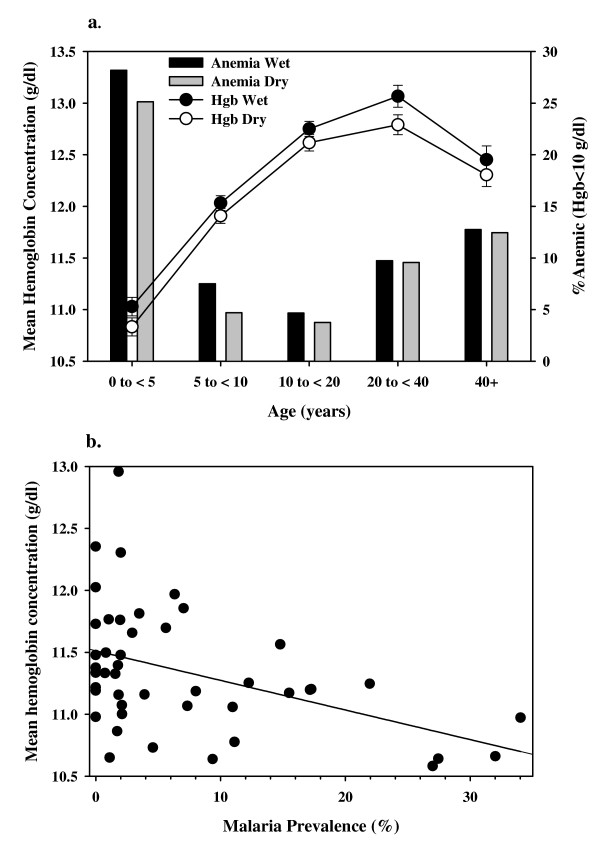
**Anaemia and malaria prevalence**. Shown are (a) the seasonal age distribution for mean haemoglobin concentration and prevalence of anaemia (hgb < 10 g/dl) in children <10 years of age and (b) the relationship, in individual sub-villages, between the overall malaria prevalence and the mean haemoglobin concentration in children under 10 years old.

In areas of high *P. falciparum *transmission in Africa, severe malarial anaemia is the most common form of severe malaria [13,14] and there is a seasonal drop in haemoglobin concentration in children during the high transmission season, probably due to increased malaria transmission [15-17]. In West Sumba, only a slight decrease in mean haemoglobin concentration in children under age 10, and an increase in the proportion of children with anaemia (hgb < 10 g/dl) in the wet season, compared to the dry season (Figure 4a), were found. Thus, at a population level, any effect of malaria transmission on haemoglobin concentration is very modest. On the other hand, the mean haemoglobin concentration was 1.1 g/dl (95% CI 0.8–1.4 g/dl) lower in parasitemic children (age < 10 years) than in non-parasitemic children. Figure 4b shows the relationship between wet season malaria prevalence in individual sub-villages, and the mean haemoglobin of children age < 10 years in those sub-villages. There is a significant association between higher malaria prevalence and lower haemoglobin concentration (P = 0.003, R^2 ^= 0.2). It is possible that this association indeed reflects reductions in haemoglobin concentration resulting from prolonged or repeated malaria infection, but it may simply reflect a poorer general health status in the sub-villages with higher malaria prevalence.

Two stage cluster sampling malaria prevalence surveys covering the West Sumba District of East Nusa Tenggara Province, Indonesia in the wet and dry seasons of 2007 were conducted. In the district as a whole malaria was hypoendemic (Table 2); the prevalence varied significantly throughout the district, and some areas in the south of the district were mesoendemic (Figure 1). *Plasmodium falciparum *was the predominant species during the wet season; in the dry season *P. falciparum *and *P. vivax *were present in roughly equal proportion (Table 2). The age distribution of malaria prevalence and parasite density (Figure 3) suggests that there has been sufficiently stable malaria transmission to induce at least partial clinical immunity; the very low malaria prevalence in children under six months is consistent with maternal immunity. Malaria infection was clearly associated with decreased haemoglobin concentrations in children (Figure 4), but it is not clear whether the association is causal. The baseline malaria prevalence data reported here provide a detailed picture of the current malaria situation in West Sumba. These data will aid the design of future control efforts and will serve as a baseline against which the results of current and future malaria control efforts can be assessed.

## Competing interests

The authors declare that they have no competing interests.

## Authors' contributions

DS and WOR designed the study, participated in data collection, analysed the data, and participated in drafting the manuscript; K, PA, S, RMD, FC, IER, IA, IE, AS, and AR participated in data collection and analysis; MK participated in data collection and analysis and wrote the first draft of the manuscript.
